# The aminoguanidine carboxylate BVT.12777 activates ATP-sensitive K^+ ^channels in the rat insulinoma cell line, CRI-G1

**DOI:** 10.1186/1471-2210-4-17

**Published:** 2004-08-24

**Authors:** Jackie M Kinsella, Hilary A Laidlaw, Teresa Tang, Jenni Harvey, Calum Sutherland, Michael LJ Ashford

**Affiliations:** 1 Division of Pathology and Neuroscience, University of Dundee, Ninewells Hospital and Medical School, Dundee DD1 9SY, UK

## Abstract

**Background:**

3-guanidinopropionic acid derivatives reduce body weight in obese, diabetic mice. We have assessed whether one of these analogues, the aminoguanidine carboxylate BVT.12777, opens K_ATP _channels in rat insulinoma cells, by the same mechanism as leptin.

**Results:**

BVT.12777 hyperpolarized CRI-G1 rat insulinoma cells by activation of K_ATP _channels. In contrast, BVT.12777 did not activate heterologously expressed pancreatic β-cell K_ATP _subunits directly. Although BVT.12777 stimulated phosphorylation of MAPK and STAT3, there was no effect on enzymes downstream of PI3K. Activation of K_ATP _in CRI-G1 cells by BVT.12777 was not dependent on MAPK or PI3K activity. Confocal imaging showed that BVT.12777 induced a re-organization of cellular actin. Furthermore, the activation of K_ATP _by BVT.12777 in CRI-G1 cells was demonstrated to be dependent on actin cytoskeletal dynamics, similar to that observed for leptin.

**Conclusions:**

This study shows that BVT.12777, like leptin, activates K_ATP _channels in insulinoma cells. Unlike leptin, BVT.12777 activates K_ATP _channels in a PI3K-independent manner, but, like leptin, channel activation is dependent on actin cytoskeleton remodelling. Thus, BVT.12777 appears to act as a leptin mimetic, at least with respect to K_ATP _channel activation, and may bypass up-stream signalling components of the leptin pathway.

## Background

ATP-sensitive K^+ ^(K_ATP_) channels are important regulators of cell function, coupling energy metabolism with electrical activity. K_ATP _channels are comprised of two proteins, derived from the sulphonylurea receptor (SUR) family and an inwardly rectifying K^+ ^channel (Kir6.x family), the exact composition of these being dependent upon tissue [1,2]. For example, pancreatic β-cells and insulin-secreting clonal cell lines express K_ATP _channels consisting of Kir6.2 and SUR1 subunits [3]. K_ATP _channels are present in numerous tissues and are the target for drugs that inhibit or increase channel activity [4,5]. The archetypal inhibitors of these channels are the sulphonylurea class of drugs, which bind to the SUR subunit of the channel. Modulation of K_ATP _channel activity in pancreatic β-cells has profound effects on insulin secretion and glucose homeostasis [6]. Sulphonylureas such as tolbutamide and glibenclamide inhibit channel activity, resulting in β-cell depolarization, increased electrical activity, enhanced calcium entry and consequently increased insulin secretion [7]. In contrast, pancreatic β-cell K_ATP _channel activation induces hyperglycaemia in animals and man [8]. This latter action is caused by membrane hyperpolarization, reduction in cell excitability and decreased intracellular calcium resulting in reduced secretion of insulin. Such effects have been reported following application of the benzothiadiazine, diazoxide, which has been used on occasion to treat persistent hyperinsulinemic hypoglycaemia of infancy [8]. It has been demonstrated that diazoxide interacts with the sulphonylurea receptor subunit, SUR1, encompassing transmembrane domains 6–11 and the first nucleotide binding fold [9]. A similar conclusion has also been reached using a novel diazoxide analogue [10]. The presence of K_ATP _channels in many other tissues, notably muscle and central neurons, has stimulated interest in the development of novel, selective K_ATP _channel openers for the treatment of various diseases [10,11].

**Figure 1:**
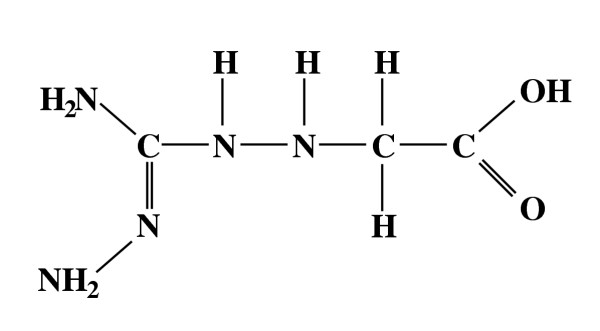
**Structure of BVT.12777 **([2-(hydrazinoiminomethyl)-hydrazino] acetic acid)

The *ob *gene product leptin has been demonstrated to activate K_ATP _channels in pancreatic β-cells [12] and insulin-secreting cell lines [13], consistent with a potential role in modifying insulin secretion [14]. One of the primary functions for this hormone is its role in the regulation of food intake and body weight [15]. Interestingly, leptin also activates K_ATP _channels of hypothalamic glucose-responsive neurones [16,17] indicating a possible role for this channel in the control of energy homeostasis and body weight. In addition, Kir6.2 knock-out mice have deficits in central glucose sensing leading to loss of glucose mediated feeding response and a defective hypoglycaemic compensatory response [18]. These latter findings suggest that hypothalamic K_ATP _channels may also be an important target for drug manipulation with respect to centrally driven control of glucose and energy homeostasis. The aminoguanidine carboxylate, BVT.12777 (Figure 1), is one of a series of structurally related molecules based on the anti-diabetic/anti-obesity agent 3-guanidinopropionic acid [19], which, like leptin, have been demonstrated to reduce body weight in obese diabetic (*ob/ob*) mice [20]. Here we demonstrate that BVT.12777 opens K_ATP _channels in the CRI-G1 insulin secreting cell line, a useful model for pancreatic β-cells [21], and for analysing the mechanism by which leptin opens K_ATP _channels [13,22,23].

## Results

### BVT.12777 activates K_ATP _channels

**Figure 2:**
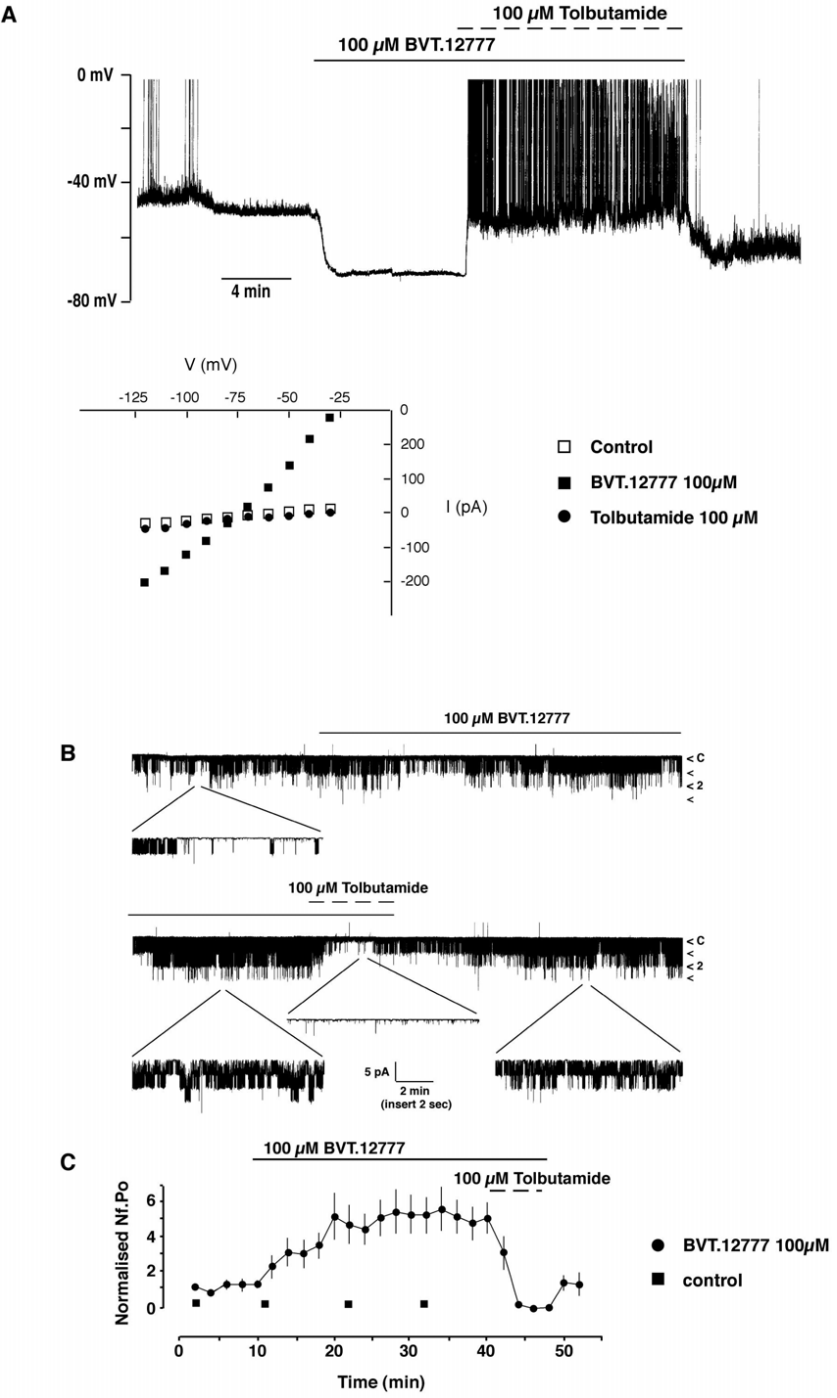
**BVT.12777 activates a tolbutamide-sensitive K+ current ***A*, the upper trace shows a current clamp recording of a CRI-G1 cell following dialysis with a 5 mM ATP-containing solution. In this and subsequent current clamp figures the trace begins approximately 5 min after formation of the whole-cell configuration. Application of BVT.12777 (100 μM) for the time indicated hyperpolarized the cell from -50 mV to -76 mV, an action readily reversed by tolbutamide (100 μM), which returned membrane potential to -54 mV. Washout of all drugs from the bath resulted in a membrane potential of -70 mV, indicating the lack of reversibility of BVT.12777. The lower plot is the current-voltage relationship for the voltage clamped currents. Cells were voltage clamped at -50 mV and 10 mV steps of 100 ms duration were applied every 200 ms (range -120 to -30 mV). BVT.12777 increased the membrane conductance relative to control and tolbutamide reversed this BVT.12777-induced conductance increase with a reversal potential of -78 mV. *B*, cell-attached recording from a CRI-G1 cell, at 10 mV applied to the recording pipette. Single channel openings are shown as downward deflections. Addition of 100 μM BVT.12777 induced an increase in channel activity (N_f_.P_o_) from 0.17 in control to 0.31, and 1.25 at 10 and 20 minutes respectively, after BVT addition. Application of 100 μM tolbutamide induced a substantial inhibition of activity (to 0.02), which was reversed on washout of all drugs, with activity increasing to 0.74. The symbol C refers to the closed state of the channel in this and subsequent figures. *C*, diary plot of N_f_.P_o _against time from cell-attached experiments in the presence and absence of BVT.12777, where channel activity was calculated every 2 minutes. Each point is the mean of 4–7 separate determinations.

Under current clamp conditions with 5 mM ATP in the pipette solution to maintain K_ATP _channels in the closed state, the mean resting potential was -38.7 ± 1.7 mV (n = 10), similar to values reported in previous studies [13,22] under these recording conditions. Application of BVT.12777 (100 μM) hyperpolarized CRI-G1 cells (Figure 2A) to -66.3 ± 2.7 mV (n = 10). Examination of the voltage-clamped macroscopic currents indicates that prior to the addition of BVT.12777 the slope conductance of the cells was 0.43 ± 0.03 nS (n = 10), and following exposure to BVT.12777 (100 μM), this increased to 3.45 ± 1.17 nS (n = 10). The reversal potential (obtained from the point of intersection of the current-voltage relationship) associated with the BVT.12777-induced conductance increase (Figure 2A) was -78.5 ± 0.8 mV (n = 10), close to the calculated value for E_k _of -84 mV in this system, indicating increased K^+ ^conductance. CRI-G1 cells responded to BVT.12777 in an all or none manner, with cells undergoing full hyperpolarization and increase in conductance, at all concentrations (100 – 300 μM) examined. Such an effect has also been reported for leptin on CRI-G1 cells [13]. Removal of BVT.12777 from the bath solution did not fully recover the membrane potential and conductance to control values over the next 15–30 minutes (not shown). Application of the K_ATP _channel inhibitor, tolbutamide (100 μM) during BVT.12777 exposure (Figure 2A) completely reversed the BVT.12777-induced hyperpolarization and decreased conductance, to -41.0 ± 4.8 mV (n = 5) and 0.58 ± 0.07 nS (n = 5) respectively, values indistinguishable from control (P > 0.05). These data indicate that BVT.12777 increases K_ATP _current in this cell line. This is demonstrated more clearly in cell-attached recordings from CRI-G1 cells, where bath application of BVT.12777 (100 μM) resulted in activation of single K_ATP _channel currents (Figure 2B; n = 7). The increase in channel activity was evident within 5 minutes of drug application, was sustained over the time course of exposure (~30 minutes) and was not immediately reversed following removal of the drug. Figure 2C shows mean channel activity (N_f_.P_o_), normalised to the control for each recording, plotted against time of exposure to BVT.12777. BVT.12777 activation of K_ATP _channels was demonstrated to be reversibly inhibited by 100 μM tolbutamide (n = 4; Figure 2B,2C). Identical control experiments, in the absence of BVT.12777, resulted in no significant effect on K_ATP _channel activity, over a 30-minute test period (n = 8; P > 0.05).

**Figure 3:**
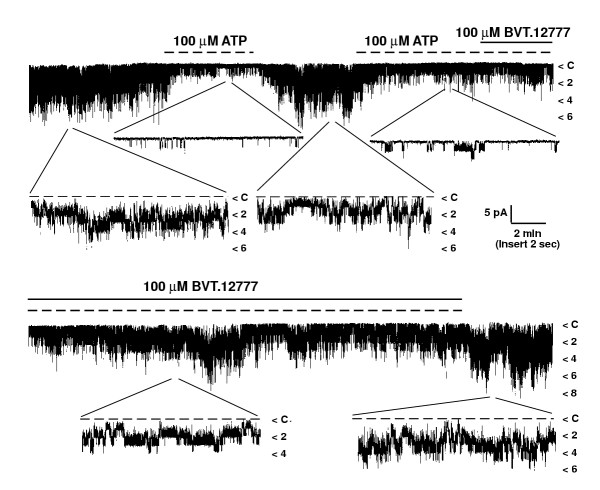
**BVT.12777 activates K_ATP _channels in inside-out patches **Continuous single channel currents recorded from an inside-out patch at a holding potential of -40 mV. Application of 100 μM MgATP reversibly inhibited channel activity by >90%, demonstrating K_ATP _identity. Addition of 100 μM BVT.12777, in the presence of 100 μM MgATP to the cytoplasmic aspect of the patch resulted in K_ATP _channel activation. N_f_.P_o _values were 2.96 (control, after first MgATP challenge), and 0.25 in the presence of MgATP, which increased to 0.72, 1.06 and 2.74 at 5, 10 and 20 minutes respectively, after BVT.12777 addition.

The effect of BVT.12777 on K_ATP _channel activity in excised membrane patches was also examined. Recordings were made from inside-out patches in symmetrical (140 mM KCl in pipette and bath solutions) K^+ ^at a membrane potential of -40 mV. K_ATP _channels were identified by inhibition of channel activity following application of 100 μM MgATP to the inner membrane aspect of the patch, which reduced normalised N_f_P_o _from 1.0 to 0.23 ± 0.05 (n = 4; P < 0.05). Subsequent application of 100 μM BVT.12777, in the continued presence of MgATP, induced a gradual increase in K_ATP _channel activity (Figure 3), to levels similar to that of control (in the absence of MgATP). For example 15 minutes after 100 μM BVT.12777 application normalised mean channel activity had recovered to 1.18 ± 0.46 (n = 4). In experiments where no drug was added, K_ATP _channel currents, in the presence of 100 μM MgATP, did not activate spontaneously (n = 4).

### BVT.12777 activates K_ATP _channels independently of PI 3-kinase activity

Leptin and diazoxide hyperpolarized CRI-G1 cells, in a manner similar to that of BVT.12777 (data not shown). Leptin (10 nM) induced a hyperpolarization from a mean membrane potential of -47.6 ± 1.6 mV to -68.5 ± 1.9 mV (n = 8; P < 0.05), and application of tolbutamide (100 μM) reversed this action, returning the membrane potential to -47.5 ± 1.9 mV (n = 4). Diazoxide (200 μM) rapidly hyperpolarized CRI-G1 cells from a mean membrane potential of -49.9 ± 1.7 mV to -74.0 ± 1.5 mV (n = 6; P < 0.05), with tolbutamide (100 μM) also reversing this action, returning membrane potential to -46.9 ± 3.8 mV (n = 6). Leptin, but not diazoxide activation of CRI-G1 K_ATP _channels is PI3K dependent [22,23]. Thus, we investigated whether BVT.12777 activates K_ATP _channels in CRI-G1 cells by direct (like diazoxide) or indirect (like leptin) mechanisms.

**Figure 4:**
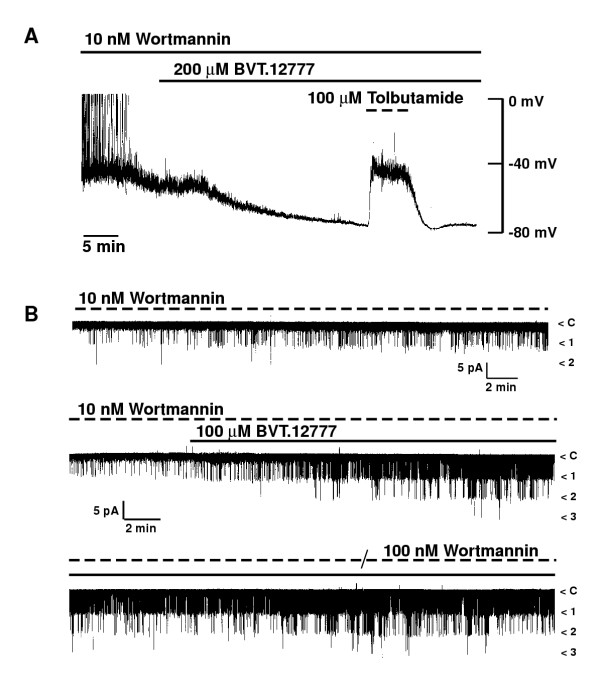
**Wortmannin does not inhibit BVT.12777 activation of K_ATP _***A*, current clamp record of a CRI-G1 cell dialysed with 5 mM MgATP, following exposure of cells to 10 nM wortmannin for 15–20 minutes. Application of BVT.12777 (200 μM), in the continued presence of wortmannin hyperpolarized the cell from -46 to -77 mV. Tolbutamide (100 μM), applied after the BVT-induced hyperpolarization, recovered the membrane potential (to -40 mV). *B*, cell-attached recordings from CRI-G1 cells, following exposure of cells to 10 nM wortmannin for 15–20 minutes. Upper trace; in the continued presence of wortmannin, N_f_.P_o _was 0.01 and 0.03 after 5 and 30 minutes respectively. Lower trace, application of BVT.12777 (100 μM) to cell-attached recording in the presence of 10 nM wortmannin resulted in K_ATP _activation, with N_f_.P_o _values of 0.01, 0.12 and 0.27 prior to, and 10 and 30 minutes after, BVT.12777, respectively. Addition of 100 nM wortmannin did not inhibit channel activity.

Pre-incubation of CRI-G1 cells (20 min) with inhibitors of PI 3-kinase, wortmannin (10 nM) or LY294002 (10 μM) had no significant effect on the mean resting membrane potential or slope conductance of CRI-G1 cells and did not prevent BVT.12777 from causing hyperpolarization and increased cell conductance (Figure 4A). In the presence of 10 nM wortmannin, values for mean membrane potential and slope conductance were -44.3 ± 1.2 mV (n = 6) and 0.86 ± 0.10 nS (n = 5), and addition of 200 μM BVT.12777 hyperpolarized cells to -68.9 ± 0.8 mV (n = 6) with an increase in slope conductance to 3.10 ± 0.38 nS (n = 5). Identical results were obtained in the presence of 10 μM LY294002 (data not shown), with corresponding control values of -40.8 ± 2.8 mV (n = 6) and 0.79 ± 0.11 nS (n = 4), and in the presence of 200 μM BVT.12777, -67.9 ± 0.6 mV (n = 6) and 2.69 ± 0.35 nS (n = 4) for membrane potential and slope conductance respectively. In all experiments (i.e with either PI3K inhibitor) addition of tolbutamide (100 μM) recovered the membrane potential (-41.8 ± 1.5 and -34.0 ± 1.7 mV; n = 6) and slope conductance (0.89 ± 0.11 (n = 5) and 0.58 ± 0.06 (n = 4) nS) for wortmannin and LY294002 respectively, to values indistinguishable from controls (P > 0.1). Cell-attached recordings from CRI-G1 cells also show that wortmannin (10 – 100 nM) did not occlude BVT.12777 activation of K_ATP _channels (Figure 4B). Mean channel activity in the presence of wortmannin (10 nM) was 0.02 ± 0.00 which increased to 0.16 ± 0.02, 20 minutes after exposure to 100 μM BVT.12777 (n = 3; P < 0.05). Control experiments where no BVT.12777 was added show no change in channel activity over a 30-minute period (N_f_.P_o _= 0.01 ± 0.00 and 0.04 ± 0.00 after 5 and 30 minutes respectively; n = 4).

### Heterologously expressed K_ATP _currents are not activated by BVT.12777

Oocytes injected with Kir6.2 and SUR1 cRNAs were challenged with sodium azide (3 mM) to elicit a reversible increase in current, which was completely blocked by 1 μM glibenclamide or 0.5 mM tolbutamide, indicating that the current was due to K_ATP _activation, as described previously [24,25]. In oocytes, previously exposed to sodium azide in order to verify Kir6.2-SUR1 expression, application of BVT.12777 (10 μM – 1 mM) did not produce any consistent increase in K_ATP _current (n = 16; data not shown). Consequently, we utilized an alternative expression system, the HEK 293 cell line [25]. Application of BVT.12777 (100 μM) to the bathing solution using the cell-attached recording configuration resulted in no significant increase in mean channel activity above control levels over a 30-minute period, although subsequent addition of sodium azide (3 mM) did cause a rapid increase in channel activity, which was reversed by the addition of 100 μM tolbutamide (n = 4, data not shown). Similarly, application of BVT.12777 in the presence of 0.1 mM MgATP to inside-out patches from HEK 293 cells transiently expressing Kir6.2-SUR1, did not cause activation of channel activity following 30 minutes exposure (n = 4; data not shown). Thus BVT.12777 does not appear to be capable of activating heterologously expressed Kir6.2-SUR1 currents.

### MAPK does not mediate BVT.12777-activation of K_ATP_

Exposure of CRI-G1 cells to BVT.12777 (100 μM) for up to 30 minutes had no consistent effect on the phosphorylation of enzymes downstream of PI3K (PKB and its downstream target, GSK3), but did increase the phosphorylation of STAT3 (n = 4) and MAPK (n = 4; data not shown). These data are in agreement with the lack of BVT.12777 sensitivity to PI3K inhibitors on activation of K_ATP _channels. However, activation of MAPK has been implicated as a significant intermediate for both insulin and leptin signalling pathways in various cell types [26-29]. Thus, we examined the effect of UO126, a potent and specific inhibitor of the activation of the classical MAPK cascade [30], on BVT.12777 opening of K_ATP _channels. Application of UO126 (25 μM) inhibited approximately 90 % of K_ATP _channel activity in cell-attached or inside-out recordings, whereas 1–10 μM UO126, concentrations that suppresses activation of MAPKK [30], had no significant effect on channel activity (data not shown). Control cell attached recordings had a mean channel activity of 0.07 ± 0.02, which increased to 1.29 ± 0.82 (n = 3) in the presence of BVT.12777 (100 μM). Subsequent application of UO126 (1 μM) in the continued presence of BVT.12777 did not alter channel activity (data not shown), over a 15-minute period (N_f_.P_o _was 1.86 ± 1.45 (n = 3) and 2.44 ± 2.00 (n = 3), at 5 and 15 minutes respectively; P < 0.05). In addition, increasing UO126 to 10 μM had no effect on BVT.12777 induced K_ATP _channel activation.

### BVT.12777 activation of K_ATP _channels is dependent on actin cytoskeleton dynamics

**Figure 5:**
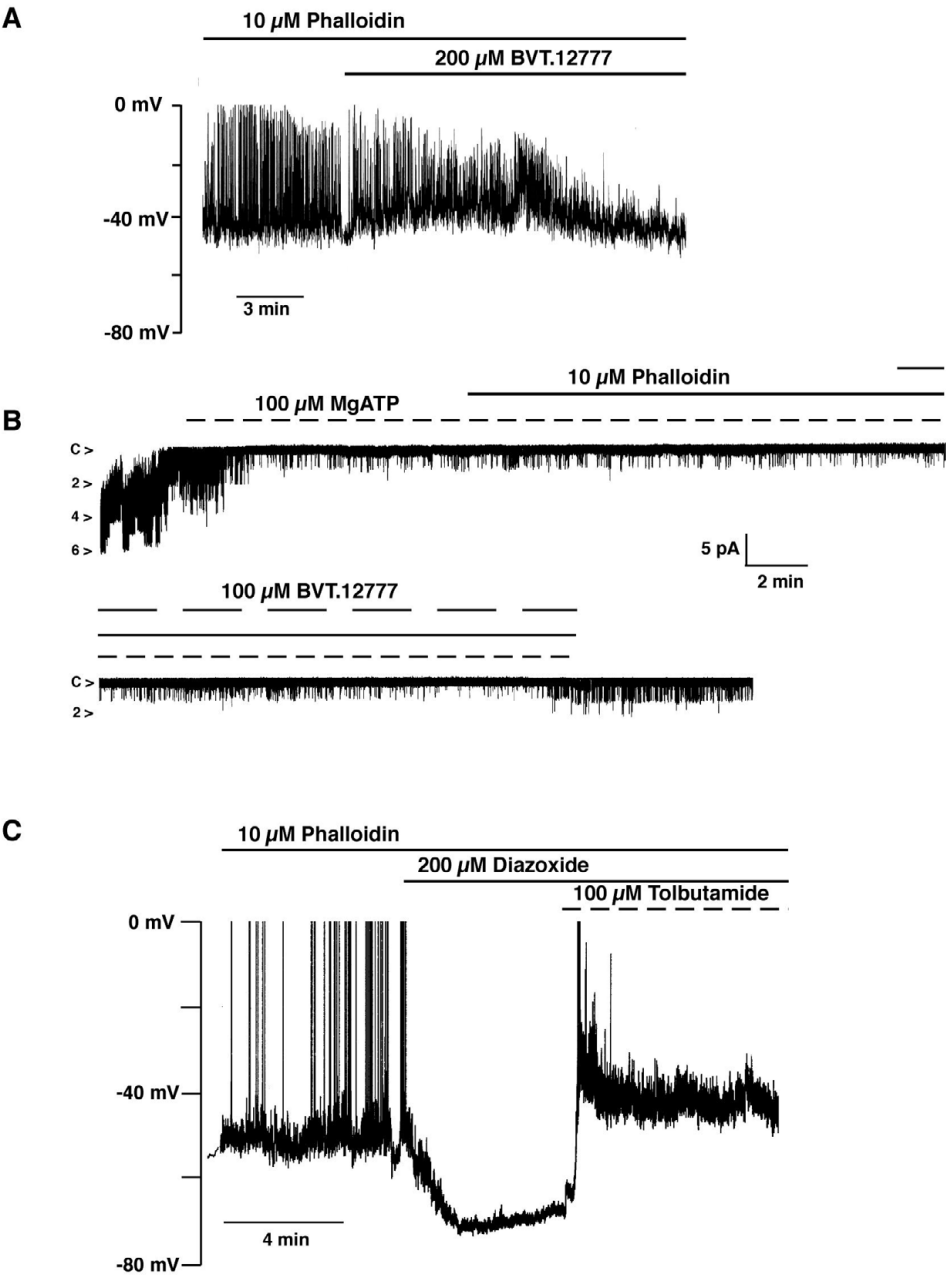
**Phalloidin prevents BVT.12777 activation of K_ATP _***A*, current clamp record of a CRI-G1 cell dialysed with 5 mM MgATP and 10 μM phalloidin. Application of BVT.12777 (200 μM) had no effect on the membrane potential of the cell (-41 mV) in the presence of phalloidin. *B*, continuous single channel currents recorded from an inside-out patch at a holding potential of -40 mV. Application of 100 μM MgATP reversibly inhibited N_f_.P_o _from 1.25 to 0.02. Addition of 10 μM phalloidin and subsequently 100 μM BVT.12777, in the presence of 100 μM MgATP, to the cytoplasmic aspect of the patch resulted in no effect on K_ATP_, with N_f_.P_o _values of 0.01 and 0.03 respectively. *C*, current clamp record of a CRI-G1 cell dialysed with 5 mM MgATP and 10 μM phalloidin. Application of diazoxide (200 μM) induced rapid cell membrane hyperpolarization, from -55 to -72 mV, an action reversed (to -45 mV) by tolbutamide (100 μM).

Leptin activation of K_ATP _channels in the CRI-G1 cell line is dependent upon reorganisation of the cytoskeleton, a process downstream from PI3K activation [31]. Therefore, we examined whether BVT.12777 opening of CRI-G1 K_ATP _channels occurs through alteration of actin filament dynamics. For this series of experiments the heptapeptide mushroom toxin phalloidin [32] was used to stabilise the polymerised form of actin (F-actin). As phalloidin is membrane-impermeant, it was directly applied to the internal aspect of the cell membrane. In whole-cell experiments, 10 μM phalloidin was added to the electrode solution and allowed to dialyse into the cell. The mean resting potential and slope conductance were -38.0 ± 0.6 mV and 0.66 ± 0.04 nS (n = 4) respectively, and following addition of 200 μM BVT.12777 no significant change in these parameters was observed (Figure 5A), with a mean membrane potential of -41.7 ± 1.1 mV and slope conductance of 0.60 ± 0.08 nS (n = 4; P > 0.05). The presence of phalloidin (10 μM) in the bath solution also prevented K_ATP _channel activation by BVT.12777 in the inside-out isolated patch configuration (Figure 5B). Application of 0.1 mM MgATP to the cytoplasmic aspect of inside-out patches caused 97.5 ± 2.1% inhibition of K_ATP _channel activity (n = 3; P < 0.05) and subsequent addition of 10 μM phalloidin had no further effect, as reported previously [29]. Subsequent addition of BVT.12777 (100 μM) failed to increase K_ATP _channel activity, with mean Nf.Po values of 0.06 ± 0.05 and 0.03 ± 0.01 in the absence and presence of BVT.12777 respectively (n = 3; P > 0.05). In contrast, the direct K_ATP _channel opener, diazoxide activates K_ATP _channels in the presence of phalloidin. In whole-cell experiments (Figure 5C), diazoxide (200 μM) hyperpolarized CRI-G1 cells from a mean membrane potential of -42.6 ± 0.1 mV to -70.1 ± 0.8 mV (n = 4; P < 0.05), and increased slope conductance from 0.87 ± 0.23 to 7.39 ± 0.72, actions reversed by tolbutamide (100 μM).

### F-actin is disrupted by BVT.12777

**Figure 6:**
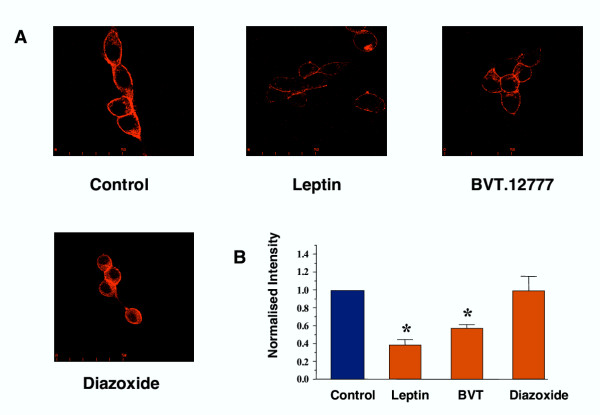
**BVT.12777 disrupts the actin cytoskeleton ***A*, images of rhodamine-conjugated phalloidin fluorescence in CRI-G1 cells in control conditions and following incubation with leptin (10 nM), BVT.12777 (100 μM) or diazoxide (200 μM) for 30 minutes. All panels show representative X-Y images. Note the marked reduction in phalloidin staining in cells pre-treated with leptin or BVT.12777, and not diazoxide. Scale bars are 50 μm. *B*, histogram comparing the normalised fluorescence intensity relative to control in the membrane periphery of randomly selected CRI-G1 cells for each condition; (control (n = 13; cells = 195), 10 nM leptin (n = 6; cells = 90), BVT.12777 (n = 6; cells = 90) and 200 μM diazoxide (n = 4, cells = 60). Error bars indicate s.e.m. and * significance of P < 0.001.

The prevention of BVT.12777-induced K_ATP _activation by phalloidin mirrors the effect of this toxin on leptin activation of K_ATP _[31]. Thus, we visualised F-actin by staining with rhodamine-conjugated phalloidin. In untreated CRI-G1 cells there was pronounced phalloidin-positive labelling of the cell membrane, with more diffuse, granular staining within the cytoplasm (Figure 6A). In contrast, cells treated with BVT.12777 (100 μM) or leptin (10 nM) for 40 min showed a marked reduction in phalloidin fluorescence intensity, with disjointed labelling at the cell membrane (Figure 6A). The actin filament disrupter cytochalasin B [33] also reduced the intensity of phalloidin labelling but in a more punctate manner on visualisation of treated cells compared with controls (data not shown). Analysis of the mean fluorescence intensity at the cell membrane following the actions of BVT.12777 and leptin demonstrated that both treatments caused a significant reduction of the intensity of rhodamine-phalloidin labelling, by 43.0 ± 4.2% (n = 6; P < 0.05) and 62.2 ± 6.0% (n = 6; P < 0.05), respectively, compared to untreated cells (Figure 6B). However, the directly acting K_ATP _channel opener, diazoxide did not cause disruption of the actin cytoskeleton (Figure 6A,6B), with a relative intensity of rhodamine-phalloidin staining of 0.98 ± 0.16 (P > 0.05).

## Discussion

BVT.12777 induced hyperpolarization of CRI-G1 cells, with an associated increase in K^+ ^conductance, an action likely caused by the activation of K_ATP _channels, as the sulphonylurea tolbutamide completely reversed its effects. Cell-attached and inside-out single channel current recordings demonstrate directly that BVT.12777 activates K_ATP _channels. The increased K_ATP _current generated in isolated membrane patches resembles the effects of K_ATP _activators such as diazoxide [34] and sodium azide [35], which have also been shown to activate insulinoma or pancreatic β-cell K_ATP _channels in isolated patches in the presence of Mg-ATP. Thus, although not tested here, BVT.12777 as an activator of K_ATP _would be expected, as observed for diazoxide, to inhibit insulin release from CRI-G1 cells stimulated by metabolizable substrates or tolbutamide [36], although this would clearly be dependent on its action on other β-cell conductances, notably calcium channels. BVT.12777 activation of K_ATP _channels was only slowly reversed on withdrawal of the drug, unlike the actions of diazoxide or sodium azide, which are rapidly reversed on washout [35,36]. Indeed, following removal of BVT.12777 in the absence or presence of tolbutamide, enhanced K_ATP _channel activity was apparent for a considerable time. The slow reversibility on washout of BVT.12777 resembles the effects of the hormone leptin on CRI-G1 cell membrane potential and K_ATP _channel activation [13].

Leptin, via activation of the main signalling form of the leptin receptor (ObRb), has been shown to increase the phosphorylation of STAT3, MAPK and to stimulate PI3K pathways in various peripheral tissues, cell lines [37], and in hypothalamic neurones [38]. BVT.12777 although stimulating phosphorylation of STAT3 and MAPK did not stimulate PI3K dependent pathways as demonstrated by the lack of effect on the phosphorylation status of the PI3K output indicators, PKB and GSK3. It is unclear at present how this molecule induces STAT3 and MAPK phosphorylation. As K_ATP _activation by BVT.12777 is rapid and occurs in isolated membrane patches it is unlikely that any JAK-STAT pathway (which drives changes in transcription) contributes to this action. Leptin activation of K_ATP _channel currents in CRI-G1 cells has previously been shown to be independent of MAPK, but prevented by the inhibitors of PI3K [22]. However, BVT.12777 activation was not only insensitive to inhibition by the MAPKK inhibitor, UO126, it was also insensitive to the presence of the PI3-kinase inhibitors, wortmannin and LY294002, at concentrations sufficient to prevent leptin activation of K_ATP _in this cell line. These data led us to suspect that BVT.12777, irrespective of its ability to initiate various signalling cascades in this cell line, increased K_ATP _channel activity by a more direct effect on the channel subunits in a manner analogous to diazoxide, which is purported to interact directly with the SUR1 subunit [9,10]. This possibility was tested by heterologous expression of the β-cell subunits of K_ATP _channels, Kir6.2 and SUR1, in Xenopus oocytes, a commonly utilised expression system for electrophysiological studies of these recombinant channels [24,25]. However, BVT.12777 did not activate Kir6.2-SUR1 currents in oocytes, demonstrated to express functional K_ATP _channel currents. Thus we explored this question further by utilising a second heterologous expression system for Kir6.2-SUR1, HEK293 cells. Recordings from inside-out patches demonstrated that BVT.12777 did not activate Kir6.2-SUR1 currents in the presence of Mg-ATP, in contrast to diazoxide [39] or sodium azide [35]. Overall these data strongly suggest that expression of the K_ATP _channel subunits, Kir6.2 and SUR1 are insufficient *per se *to bring about sensitivity to BVT.12777, and indicate that this opener may activate this channel type by an indirect mechanism (which is not available in oocytes or HEK cells).

Although the activation of K_ATP _channels by leptin in CRI-G1 cells is PI3-kinase dependent the lipid products of this enzyme system, such as PtdIns(3,4,5)P_3 _also do not interact directly with K_ATP _channels [22]. Recent studies demonstrate that both leptin and PtdIns(3,4,5)P_3 _increase K_ATP _channel activity indirectly, through changes in cytoskeletal dynamics [31]. It is well established that many ion channels and transporters are anchored in the membrane by either direct or indirect association with the cytoskeleton. In addition, there is growing evidence that altering the integrity of cytoskeletal elements, in particular actin filaments, can modulate the activity of a variety of ion channels [40] and receptors [41]. For example, disruption of actin filaments with cytochalasin is shown to increase K_ATP _channel activity in cardiac myocytes [42] and CRI-G1 cells [31]. Indeed, a number of lipid kinases, including PI 3-kinase, are also localised to the cytoskeleton and their activities are modulated by a variety of cytoskeletal proteins, especially those associated with actin [40]. Actin filament structure is controlled by reversible polymerisation of G-actin, which forms F-actin, and this process is under the dynamic control of various actin-binding proteins [43]. The heptapeptide mushroom toxin phalloidin [32] binds to filamentous F-actin with high affinity and stabilises the actin in this form. The addition of phalloidin to the intracellular aspect of CRI-G1 cells prevented BVT.12777, but not diazoxide, from activation of K_ATP _channel currents in whole cell and inside out recording configurations indicating that this molecule likely causes the opening of K_ATP _channels by a membrane delimited alteration of cytoskeletal dynamics. This mechanism of action is identical to that proposed for leptin and PtdIns(3,4,5)P_3 _activation of K_ATP _in this cell line [31]. Fluorescence staining of CRI-G1 cells with rhodamine-conjugated phalloidin revealed disassembly of actin filaments by both BVT.12777 and leptin, but not diazoxide. These data provide direct support for an important role for cytoskeletal dynamics in the control of K_ATP _channel activity by both leptin and BVT.12777. The lack of effect of diazoxide on the actin filament structure is also supportive of this opener acting directly on the K_ATP _channel subunits.

## Conclusions

BVT.12777 activation of K_ATP _channels in CRI-G1 cells was evident regardless of whether it was applied to the external or internal surface of the cell. BVT.12777 signalling to K_ATP _channels is not mediated by PI 3-kinase or MAPK, but does appear to depend on actin filament re-modelling. As leptin hyperpolarizes a sub-population of hypothalamic neurones by opening K_ATP _channels [16], it is feasible that at least part of the anti-obesity action of BVT.12777 may be through the activation of this potassium channel. Furthermore, as BVT.12777 acts downstream of PI3K, such an agent may act to overcome the putative central leptin resistance associated with the obese state [37]. Thus, although BVT.12777 and its close structural analogues are unlikely *per se *to be useful anti-obesity agents as they display hepatotoxicity [44], understanding the general principles underlying their mechanism of action may reveal clues for future anti-obesity drug development.

## Methods

### Cell culture and transfection

Cells from the insulin secreting cell line, CRI-G1, and the human embryonic kidney cell line, HEK 293, were grown as described previously [25,35]. The preparation of mouse Kir6.2 (provided by Professor F. Ashcroft, University of Oxford), rat SUR 1 (provided by Dr G. Bell, University of Chicago) and CD4 cDNAs and transfection procedures were as described by [25]. Transfected cells were selected by visible binding of anti-CD4 coated beads (Dynal, Oslo) following incubation with the beads for 20 min.

### Oocyte collection and preparation

Ovarian lobes were removed from mature female *Xenopus laevis *frogs (Blades Biological, UK) following killing of the animal by destruction of the brain. The use of animals was in accordance with the Home Office Animals (Scientific Procedures) Act (1986) and approved by the local ethics committee. Separation and selection of oocytes and the preparation and injection of cRNAs were performed as described by [25].

### Western blotting

CRI-G1 cells, in normal saline (containing in mM; NaCl 135, KCl 5, MgCl_2 _1, CaCl_2_, 1, HEPES 10 with glucose 10 (pH 7.4) were treated with BVT.12777 (100 μM) for 0, 1, 5, 15 or 30 minutes and whole-cell extracts were prepared as described [23]. Proteins (10 μg) were suspended in loading buffer (Invitrogen) and after denaturation, loaded on to NuPage 4–12% Bis-Tris mini-gels (Invitrogen) and run at 200 V for 1 hr. Subsequently, proteins were transferred to Hybond-C Extra nitrocellulose membranes (Amersham) at 25 V for 80 minutes at room temperature. Membranes were incubated in blocking buffer (5% non-fat milk in TBST (20 mM Tris HCl, 150 mM NaCl, 0.5% Tween, pH 7.4)) for 1 hr at room temperature after which antibodies to phospho-MAPK, phospho-STAT3, phospho PKB, phospho-GSK3 and PKB (all at 1:1000) were applied at 4°C with gentle shaking, overnight. The membranes were washed with TBST (4 × 30 minutes) and incubated for 1 hr at room temperature with HRP conjugated ImmunoPure goat anti-rabbit IgG (1:5000). After washing with TBST (5 × 15 minutes), immunoreactive bands were visualised by the enhanced chemiluminescence (ECL) detection reagent (Amersham).

### Cytoskeletal fluorescence imaging and analysis

CRI-G1 cells were gently washed in normal saline (containing in mM): NaCl 135, KCl 5, MgCl_2 _1, CaCl_2 _1, HEPES 10, pH 7.4, and incubated for 40 min with either 100 μM BVT.12777, 10 nM leptin, 200 μM diazoxide or 3 mM sodium azide for 30 min with the cytoskeletal disrupter, cytochalasin B (10 μM). Cells were then fixed, permeabilised, stained with rhodamine-conjugated phalloidin (2.66 U ml^-1^) and visualised using a BioRad Microradiance, confocal imaging system as described by [31]. The intensity of rhodamine-conjugated phalloidin staining in the plasma membrane was determined using BioRad Lasersharp processing software (Bio-Rad, CA, USA). Analysis lines were drawn along randomly selected regions of the plasma membrane and the fluorescence intensity determined. A histogram giving the mean fluorescence intensity was constructed for a minimum of 5 cells on each stimulated or control dish on at least 3 separate occasions. Within a given experimental series all conditions for capturing images were constant. In order to allow for quantification of experimental data obtained on separate days, the results were normalised relative to the mean plasma membrane fluorescence measured in the control cells for each day and presented as mean ± S.E.M. Statistical analyses were performed using Student's unpaired *t *test. p < 0.05 was considered significant.

### Electrophysiological recording and analysis

Whole cell currents from Xenopus oocytes were measured using a two-electrode voltage clamp technique as described by [25]. Recordings were made in a high-potassium bath solution, KD96 containing (mM): KCl 96, NaCl 2, CaCl_2 _1.8, HEPES 5 (pH 7.4 with KOH). Working concentrations of drugs were prepared in KD96 and superfused into the bath. Whole-cell current-clamp recordings with excursions to voltage clamp mode were used to monitor membrane potential and macroscopic currents from CRI-G1 cells. Cell-attached and excised inside-out recordings were made from CRI-G1 cells and HEK cells expressing Kir6.2 and SUR1 to examine single channel responses as described previously [25,35]. Single channel data were analysed for current amplitude and channel activity (N_f_.P_o_; where N_f _is the number of functional channels in the patch and P_o _is the open probability) as described previously [45]. All data were normalised to control and are expressed as mean ± S.E.M. Statistical analyses were performed using Student's unpaired *t *test. P < 0.05 was considered significant. Recording electrodes were pulled from borosilicate glass and had resistances of 2–5 MΩ for whole cell recordings and 7–10 MΩ for cell-attached and inside-out experiments when filled with electrolyte solution. The pipette solution for whole-cell recordings comprised (in mM): KCl 140, MgCl_2 _0.6, CaCl_2 _2.73, Mg-ATP 5.0, EGTA 10, HEPES 10, pH 7.2 (free [Ca^2+^] of 100 nM), whereas for single channel recordings the pipette solution contained (in mM): KCl 140, CaCl_2 _1, MgCl_2 _1, HEPES 10, pH 7.2. The bath solution for whole-cell and cell-attached recordings was normal saline whereas for inside-out patches the bath solution contained (in mM): KCl 140, MgCl_2 _1, CaCl_2 _2, EGTA 10, HEPES 10, pH 7.2 (free [Ca^2+^] of 30 nM). All solution changes were achieved by superfusing the bath with a gravity feed system at a rate of 10 ml min^-1^, which allowed complete exchange within 2 min. All experiments were performed at room temperature (22–25°C).

### Antibodies & drugs

Anti-PKB, which recognises all three isoforms of PKB, and the phospho-specific PKB (Thr308), GSK3α/β (Ser21/9), STAT3 (Tyr705) and p44/42 MAPK (Thr202/Tyr204) antibodies were obtained from Cell Signalling Technology Inc. Recombinant human leptin, wortmannin and LY 294002 were obtained from Novachem-Calbiochem and BVT.12777 ([2-(hydrazinoiminomethyl) hydrazino] acetic acid) was a gift from Biovitrum (Stockholm, Sweden). Tolbutamide, Mg-ATP, diazoxide, sodium azide, phalloidin and cytochalasin B were obtained from Sigma. Rhodamine-conjugated phalloidin was obtained from Molecular Probes and UO126 from Promega. BVT.12777 was prepared as a 100 mM stock solution in normal saline and stored at -70°C prior to use. Leptin was prepared as a 10 μM stock solution in normal saline containing 0.2 % bovine serum albumin as carrier. Rhodamine-conjugated phalloidin (200 U ml^-1^) and LY 294002 (10 mM) were stored as stock solutions in 1% methanol at -20°C. Cytochalasin B was stored as a 10 mM stock solution, and diazoxide and tolbutamide as 100 mM solutions, all in DMSO at 2–4°C. Mg-ATP was stored at -20°C as a 100 mM solution in 10 mM HEPES (pH 7.2). Wortmannin and UO126 were stored as 10 mM stock solutions in Me_2_SO at -20°C.

## List of abbreviations used

CRI-G1, Cambridge Rat Insulinoma-G1; GSK3, glycogen synthase kinase-3; HEK293, human embryonic kidney 293; JAK, janus kinase; K_ATP_, ATP-sensitive potassium; Kir6.2, potassium channel inward rectifier-6.2; MAPK, p42, p44 mitogen-activated protein kinase; MAPKK, MAPK kinase; ObRb, Obese (leptin) receptor-b; PKB, protein kinase B; PI3K, phosphatidylinositol 3-kinase; PtdIns(3,4,5)P_3_, phosphatidylinositol 3,4,5 tris-phosphate; STAT3, signal transducer and activator of transcription-3; SUR, sulphonylurea receptor

## Authors' contributions

JK carried out the majority of the electrophysiology and cytoskeletal fluorescence studies. HL carried out the western blot experiments. TT and JH participated in the electrophysiological experiments. CS participated in the design and implementation of the western blot experiments. MA conceived of the study, participated in its design and co-ordination and drafted the manuscript. All authors read and approved the final manuscript.
